# BFL-1 expression determines the efficacy of venetoclax in MYC+/BCL2+ double hit lymphoma

**DOI:** 10.18632/oncoscience.402

**Published:** 2018-04-29

**Authors:** Anna Esteve-Arenys, Gael Roue

**Affiliations:** Laboratory of Experimental Hematology, VHIO, Vall d'Hebron University Hospital, C/Natzaret, 115-117 08035 Barcelona, Spain

**Keywords:** BCL-2 antagonist, bromodomain, aggressive B-cell lymphoma, drug combination, mouse model

The appropriate balance between pro-survival and pro-death BCL-2 family members in healthy cells is often disrupted in malignant B cell malignancies, where an overexpression of anti-apoptotic BCL-2 proteins can promote oncogenesis and confer resistance to chemotherapeutic agents. BH3 mimetics are the first class of drugs to target the core of the apoptosis pathway by binding directly and specifically inhibiting the anti-apoptotic members of the BCL-2 family [1]. Among this drugs, the first-in-class BCL-2-specific BH3 mimetic venetoclax (ABT-199), recently approved the treatment of patients with relapsed/refractory (R/R) chronic lymphocytic leukemia with del(17p), has also demonstrated high response rates and good toxicity profiles in other subtypes of R/R B-cell non- Hodgkin lymphoma (NHL). However, a major hurdle to its successful application is the rise of primary and acquired resistance, which pushes for the search of new therapeutic approaches. In this sense, targeted inhibition of BCL-2 is likely to have a greater clinical impact when it is combined with other agents, and a number of studies are underway to assess the safety and efficacy of combining venetoclax with standard chemotherapy (NCT03064867, NCT03054896), monoclonal antibodies (NCT03136497, NCT03135262), B-cell receptor (BCR) signaling inhibitors (NCT02756897, NCT02956382, NCT03112174) or proteasome inhibitors (NCT02755597, NCT02899052), in NHL patients [2].

High-grade B-cell lymphoma with MYC and BCL2 rearrangements (the so-called “double hit” lymphoma, DHL) is an aggressive disease characterized by frequent failures of standard chemotherapeutic regimens. First-line treatment of DHL patients is based on immunochemotherapy and intensive treatment strategies, but it does not produce a sustained remission in the majority of the cases [3]. While short-time exposure to venetoclax can trigger significant antitumoral effect in DHL cultures [4], we recently reported that prolonged exposure to the compound was associated with a decreased response of DHL cell lines at all the doses used [5]. This phenomenon was not seen in cell lines of B-cell germinal center (GCB)-diffuse large B cell lymphoma (DLBCL) origin with no concurrent MYC and BCL-2, suggesting differential compensatory mechanisms between the two NHL subtypes. The venetoclax-resistance phenotype of DHL cells was associated neither with a defective mechanism of action of the compound, as it was able to displace BIM from BCL-2 complexes in all the cell lines studied, nor with a differential basal expression of BCL-2 or BCL-2-like proteins (MCL-1, BCL-XL, BFL1/A1). Rather, and in agreement with the emerging concept that BH3 mimetic resistance is related to the upregulation of MCL-1 and/or BFL1 [6], we found BFL- 1 to be overexpressed in DHL cell lines and primary DHL cultures after prolonged exposure to venetoclax. The same observation was made in a mouse xenotransplant model of DHL after a 2 week oral treatment with the BH3 mimetic. Confirming the role of BFL-1 in acquired resistance to the drug, a standard GCB-DLBCL cell line genetically modified to overexpress this factor also showed a consistent loss of response to BCL-2 antagonism.

BFL-1 is an anti-apoptotic protein that exerts its function by sequestering pro-apoptotic/BH3-only proteins, mainly BIM, BID, PUMA, and NOXA. Its role is particularly relevant in the hematopoietic system, where it seems to be a critical downstream components of tonic and antigen-driven BCR activation, and to be involved in the lack of sensitivity of malignant B cells to chemotherapy [7]. So far, very few specific and potent inhibitors of BFL-1 have been described. Instead, indirect strategies including epigenetic drugs have been investigated. Among these latest, the preclinical evaluation of bromodomain and extra-terminal (BET) protein inhibitor, has recently offered numerous mechanistic insights in several cancer models. Within the different structure/activity-based BET protein bromodomain antagonists recently developed, JQ1 displaces the BET family member BRD4 from acetylated chromatin, resulting in the repression of MYC transcriptional program among other effects. This feature sounded particularly interesting for us, as BFL- 1 has recently been identified as a possible MYC target [8]. In our study, we employed CPI203 (Constellation Pharmaceuticals, Inc.), a JQ1-structurally related molecule with similar toxicity spectrum but with improved bioavailability profile in mice, in combination with venetoclax. Our data showed a time- and dose-dependent cytostatic effect of this drug in DHL cell lines and primary samples, which was associated with the blockade of BFL- 1 transcription and the upregulation of the pro-apoptotic gene BIM, allowing CPI203 treatment to overcome venetoclax resistance in vitro and to significantly reduce tumor burden in vivo, in agreement with preliminary results obtained with JQ1 [4].

Mechanistically, immunoprecipitation analysis suggested that the events downstream transcriptional normalization by CPI203 include the redistribution of BIM protein from BFL-1- to BCL-2-dependent complexes, and the consequent triggering of apoptotic signaling in cells exposed to venetoclax. Thus, by increasing the disbalance between intracellular pools of BIM and BFL- 1, we introduced there a clear rationale explaining the high synergistic interaction between the BCL-2 antagonist and the BET inhibitor in preclinical models of DHL.

**Figure 1:**
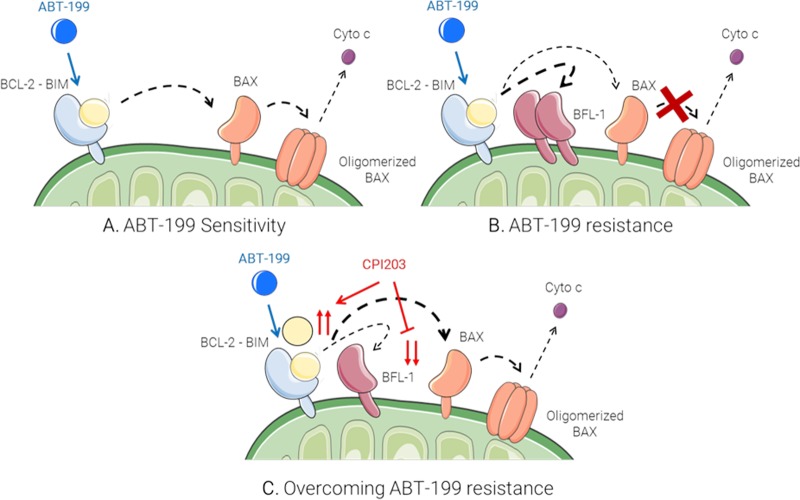
Possible mechanism of DHL cell priming by CPI203 and sensitization to venetoclax (ABT-199).

In summary, we proposed a model of DHL resistance to venetoclax in which a compensatory accumulation of BFL-1 (rather than MCL-1 or BCL-XL) can bind and inactivate the pool of BIM proteins released from BCL-2, avoiding mitochondrial depolarization and preserving cell survival after prolonged exposure to the BH3 mimetic. Regulation of DHL transcriptome by means of a small molecule BET inhibitor like CPI203 could help to circumventing this issue, allowing to the modulation of BFL1/BIM ratio, and to the priming of cells to death (Figure 1).
